# Dose- and substrate-dependent reduction of enteric methane and ammonia by natural additives *in vitro*

**DOI:** 10.3389/fvets.2023.1302346

**Published:** 2023-11-06

**Authors:** Marco Battelli, Mette Olaf Nielsen, Natalja P. Nørskov

**Affiliations:** ^1^Department of Agricultural and Environmental Sciences – Production, Landscape, Agroenergy, University of Milan, Milan, Italy; ^2^Department of Animal and Veterinary Sciences, AU Viborg – Research Center Foulum, Aarhus University, Tjele, Denmark

**Keywords:** rumen fermentation, plant secondary metabolites, quercetin, catechin, salicylic acid, tannic acid

## Abstract

Ruminants contribute to global warming by emitting greenhouse gasses, particularly methane (CH_4_) which is a product of rumen fermentation. The use of feed additives able to modulate rumen fermentation is a promising strategy to reduce enteric CH_4_ and ammonia (NH_3_) emissions. Among the various strategies investigated, plant secondary metabolites (PSMs) have attracted attention due to their apparent potential to reduce enteric CH_4_ and NH_3_ emissions, and it would be possible to use such compounds as feed additives in organic production systems. In an *in vitro* system simulating rumen fermentation, we have tested the impact of different classes of naturally occurring PSMs; catechin and quercetin (flavonoids), salicylic acid (phenolic acid) and tannic acid (hydrolysable tannin). The PSMs were added to two different basal feeds (maize and grass silages) at three inclusion doses 1.5, 3 and 6% of the feed dry matter (DM). CH_4_ production was significantly lowered upon addition of quercetin to two basal feeds at doses of 3 and 6%, and this without changes in concentrations of total volatile fatty acid (VFA) produced during fermentation. Quercetin, as the only tested additive, reduced CH_4_ production, and when added to maize silage and grass silage, the reduction increased linearly with increasing dose, ie., by 51 and 43%, respectively, at a dose of 3% of feed DM and by 86 and 58%, respectively, at a dose of 6% of feed DM. Moreover, quercetin significantly reduced NH_3_ concentration by >12% at doses of 3 and 6% in feed DM irrespective of the basal feed used as compared to when the basal feeds were incubated alone. Although none of the other additives affected CH_4_ formation, several additives had significant impacts on concentrations of NH_3_ and VFAs in the incubated fluid after fermentation. This study demonstrated a dose-dependent ability of quercetin to reduce CH_4_ emission from rumen fermentation, however, the magnitude of the suppression of CH_4_ depended on the basal feed. Furthermore, quercetin reduced NH_3_ concentration irrespective of the basal feed type. These findings encourage to *in vivo* studies to verify whether quercetin can reduce CH_4_ emission also in cows.

## 1. Introduction

Ruminants are responsible for two-thirds of the global emission of anthropogenic greenhouse gasses (GHG) imputed to the livestock sector, which represents 14.5% of the total agricultural emissions (1). Methane (CH_4_) is the major GHG formed in the rumen, where it is synthesized from hydrogen and carbon dioxide (CO_2_) formed during microbial fermentation of the feed (2, 3). CH_4_ emission represents an energy loss of 6–12% of the animal’s gross energy intake (4). Ammonia (NH_3_) emission from manure, derived from nitrogen excretion in feces and urine, represents another important emission from livestock production. To ensure a sustainable development of the livestock sector, it is crucial that these emissions are reduced dramatically in the future in view of estimated increased global demands for meat and milk (5). Several strategies have been proposed to reduce environmental impact in ruminants farming, such as increasing animal productivity, genetic selection, diet formulation or modifying rumen fermentation patterns (6). Considering this, feed additives can play a role not only in reducing the environmental impact of ruminants, but also by increasing animal health and productive performance (7, 8). The use of natural feed additives able to modulate rumen fermentation patterns is a promising strategy to reduce enteric CH_4_ and NH_3_ emissions. In this context, plant secondary metabolites (PSM) have attracted attention due to their potential to reduce enteric CH_4_ and NH_3_ emissions while improving the health status and thereby productivity of the animal (9). Since PSMs are natural products, they also represent a promising strategy to reduce emissions for organic farmers. Tannins belongs to the class of PSMs that has been most extensively studied with well documented effects on NH_3_ reduction and there is some documentation that some may also reduce CH_4_ emission (6, 10, 11). A common feature of tannins is the ability to bind proteins, forming feed complexes that are undegradable in the rumen (12). This protective effect is thought to cause lowering of the ruminal NH_3_ concentration and increased ruminal escape of dietary proteins (13). At the low pH in the abomasum, the protein-tannin complexes are subsequently dispersed, making feed proteins available again for enzymatic digestion. To a lesser extent, tannins are able to form complexes also with other components, such as carbohydrates and metal ions (14). The formation of such complexes can lead to a reduction of the overall ruminal feed degradability (15). Martínez et al. (16) tested the effect *in vitro* of adding tannic acid (TAN) to two different substrates, corn and wheat grain, to study the importance of starch structure on formation of starch-tannin complexes. At an inclusion dose of 5% (w/w DM), TAN decreased the Total Gas Production (TGP) over the first 24 h of incubation in buffered rumen fluid inoculum, but not later, when the substrate was wheat, whereas the reduction was significant throughout a 48 h incubation period with corn grain as the substrate. The differential effect of TAN depending on the nature of the basal feed was considered to be a consequence of the different architecture of the endosperm affecting the affinity of starch to tannins (16).

*In vitro* (17) and *in vivo* (18) studies have demonstrated that TAN can also reduce enteric CH_4_ emission in a dose dependent manner. In the *in vivo* study, TAN induced a dose-dependent reduction of CH_4_ emission from 11 to 33.6% when the inclusion in diets for beef cattle was increased from 0.65 to 2.6% (w/w DM) (18).

Another class of PSMs that also contain potential rumen anti-methanogens is flavonoids as suggested by findings in a few *in vitro* studies. Oskoueian et al. (19) investigated the effects of the flavonoids quercetin (QUE), catechin (CAT) as well as other PSMs at an inclusion dose of 4.5% (w/w DM), and found that QUE significantly decreased CH_4_ formation during fermentation of a feed, while CAT did not. However, in another study, CAT was shown to act as a hydrogen sink and could thereby have the potential to reduce CH_4_ (20). In a previous *in vitro* study in our laboratory, we detected a significant CH_4_ reduction by QUE, TAN and salicylic acid (SALA), whereas CAT only showed a tendency to reduce CH_4_ (21). The conflicting results and the lack of response in some *in vitro* studies (22, 23) could possibly be due to differences in dosing or in the type of feed substrate used in incubations. The present study was based on the hypotheses that the four PSMs TAN, SALA, QUE, and CAT can reduce CH_4_ production in a dose-dependent manner without negatively affecting ruminal fermentation of the feed, but the CH_4_ reducing potency of these PSMs depends on the type of feed substrate.

Therefore, the aim of this study was to establish the effects *in vitro* in a system simulating rumen fermentation of adding increasing doses (0, 1.5, 3 and 6% w/w DM) of the four PSMs to two different basal feeds, maize (MS) and grass (GS) silages with different starch and fiber composition on TGP, CH_4_ production and rumen fermentation patterns (VFA and NH_3_ concentrations).

## 2. Materials and methods

### 2.1. Chemicals

Quercetin (117-39-5), catechin hydrate (225937-10-0; DM: 97.3%), salicylic acid (69-72-7), and tannic acid (1401-55-4), were purchased from Sigma-Aldrich (Merck KGaA, Darmstadt, Germany).

All compounds were purchased as a dry powder and 0.030 g of each compound was weighed off and dissolved in 2 mL of either dimethyl sulfoxide (DMSO; Sigma) or pure water to reach the concentration of 15 mg/mL and further diluted to 7.5 and 3.75 mg/mL. Due to pour solubility of CAT, QUE, and SALA in water, these compounds were dissolved in DMSO, while pure water was used to dissolve TAN. The detailed protocol of the procedure can be found in Nørskov et al. (21).

### 2.2. *In vitro* simulation of rumen fermentation

Two commercial GP apparatuses (Ankom^RF^ GP System, Ankom Technology^®^, NY, United States) consisting each of 50 Duran^®^ bottles (capacity: 132 ± 1.1 mL) equipped with pressure sensors and wireless connection to a computer were used to test the effect of CAT, QUE, SALA, and TAN on *in vitro* rumen fermentation. To evaluate a possible interaction between the compounds and the substrate, two different basal feeds, maize silage (MS) and grass silage (GS), were used. Four experimental runs, two per each substrate, were conducted. The four compounds were tested at three levels of inclusion (1.5, 3, and 6 of feed DM) against a negative control (CTR), consisting only of one of the two basal feeds (MS-CTR and GS-CTR, respectively). All the treatments (4 compounds × 3 levels of inclusion × 2 basal feeds), the 2 CTR, and the blank (containing only the fermentation medium) were tested in triplicate per each experimental run.

On the morning of each experiment, rumen fluid was collected half an hour before morning feeding from three rumen cannulated non-pregnant dry Holstein cows housed at the experimental facility at Aarhus University, Foulum, Denmark. The handling and care of the cows complied with the guidelines set out by the Danish Ministry of Environment and Food (Act No. 2028, 2020) with respect to animal experimentation and care of animals under studies. The cows were fed at maintenance level with a standard diet composed of straw, hay, and a concentrate mixture (24). The rumen fluid was immediately transferred to preheated thermo bottles and transported to the laboratory within 30 min after sampling, where it was filtered through two layers of moist cheesecloth. For each cow, the pH of the filtrated rumen fluid was measured. The filtered rumen fluid was then mixed with a buffer solution redox indicator, reducing agent, buffer, and macro- and micro-mineral solutions as described by Menke and Steingass (25), in 2 buffer solution and 1 rumen fluid ratio, for the preparation of the fermentation medium. During preparation, the buffer solution and the fermentation medium were continuously flushed with N_2_ to maintain anaerobic conditions.

Incubations were conducted in the Duran^®^ bottles containing 0.5 g of MS or GS, 90 mL of buffered rumen fluid with or without 2 mL solution of PSM, in order to reach the concentration of 1.5, 3 and 6% (w/w) of PSM on DM basis.

The *in vitro* incubations were performed as described in details by Thorsteinsson et al. (26). The gas produced during fermentation and released from the GP apparatus was continuously collected in a gas-tight 1 L Aluminum Bag CEK-1 (GL Sciences Inc., Tokyo, Japan), attached to each module. After 24 h of incubation the gas-tight aluminum bags were removed. Ten milliliters of gas was extracted from each gas-bag using a gastight syringe with a twist valve (Hamilton Bonaduz AG, 7402 Bonaduz, Switzerland). The gas samples were transferred into evacuated gas chromatography (GC) vials (Labco Limited, Ceredigion, United Kingdom) for later CH_4_ analyses. After 48 h of incubation the bottles were put into ice bath to stop the fermentation. All the content of the bottles was filtered through F57 fiber bags (ANKOM Technology, Macedon, NY, United States) (pore size: 25 μm) and an aliquot of the filtered liquid sample was collected for VFA and NH_3_ analyses.

### 2.3. CH_4_ and VFA analyses using GC-TCD

CH_4_ concentrations in gas samples were analyzed using a Trace 1,310 GC equipped with Rt^®^-Q-BOND column, 30 m length, ID 0.25 mm and 8 μm film thickness (Restec, Bellefonte, PA, United States), TCD detector and a TriPlus Headspace autosampler (Thermo Fisher Scientific, Waltham, MA, United States), as described by Jensen et al. (27).

VFA analyses were performed as described by Olijhoek et al. (28) using a Trace 1,310 GC equipped with a 30 m × 0.53 mm × 1 μm HP-FFAP column (Agilent Technologies Inc.).

### 2.4. Chemical composition of the standard feed and chemical analyses

The MS used had the following chemical composition: organic matter (OM), 965; neutral detergent fiber, expressed exclusive of residual insoluble ash (aNDFom), 329; crude protein (CP), 77.7; starch, 351 g/kg DM.

The GS used had the following chemical composition: OM, 908; aNDFom, 360; CP, 179 g/kg DM.

The DM content of undegraded feed residues in fiber bags was determined by oven drying at 103°C overnight [AOAC (29); method 935.29], the aNDFom was analyzed following the procedure reported by Mertens (30), with the inclusion of heat-stable α-amylase and sodium sulfite, while the ash was determined by combustion at 525°C for 6 h [AOAC (29); method 942.05].

The NH_3_ concentration was determined using a Randox AM 1015 kit (Randox Laboratories, United Kingdom) and an ADVIA 1800^®^ Chemistry System (Siemens Medical Solutions, Tarrytown, NY 10591, United States) autoanalyser.

### 2.5. Statistical analyses and calculations

The cumulative gas production (psi) data recorded during the 48 h of incubation were converted into volume (mL) of gas produced at standard temperature (0°C) and pressure (1 bar) using the ideal gas law. TGP was blank corrected before the statistical analyses. The volume of CH_4_ and CO_2_ (mL) produced were calculated multiplying their concentrations (%) in the collected gas with the TGP (mL). Means of three replicates (analytical replicates) within each run were used for the statistical analysis.

The data of the various response parameters (TGP, CH_4_, CO_2_, dDM, dNDF, VFA, and NH_3_) were statistically analyzed by the mixed procedure of SAS 9.4 (SAS Institute Inc.). Initially, to test the effect of the feed and a possible interaction between the type of substrate and the type of additive, the model was:


$$Y_{\text{ijkz}}=\mu +T_{i}+L_{j}\left(T\right)+F_{k}+TxF+R_{z}+e_{\text{ijkz}}$$

where *Y_ijkz_* is the dependent response variable, *μ* is the overall mean, *T_i_* is the fixed effect of treatment (*i* = MS, GS, CAT, QUE, SALA, and TAN), *L_j_(T)* is the dose effect within the treatment (*j* = 0, 1.5, 3, 6%), *F_k_* is the fixed effect of the feed (*k* = MS, GS), *TxF* is the effect of the interaction between the treatment and the feed, *R* is the random effect of experimental run (*z* = 1, 2), *e_ijkz_* is the residual error.

Subsequently, the statistical analysis was performed separately for each type of substrate, with the following model:


$$Y_{ijz}=\mu +T_{i}+L_{j}\left(T\right)+R_{z}+e_{ijz}$$

Differences between least square means of the treatments were evaluated using Tukey’s method for comparison.

In order to evaluate the linear and the quadratic effects of the level of inclusion of each additive within the two types of substrates, matrix coefficients were generated by using the IML procedure of SAS 9.4 for unequally spaced contrasts.

The data were tested for normality of the residuals by using the Shapiro–Wilk test. Homogeneity of the variance was tested by using Bartlett’s test. For all statistical analyses, significance was declared at *p* ≤ 0.05 and trend at 0.05 < *p* ≤ 0.10. Data in the tables are presented as least squares means and standard errors. To make the table simpler to read and avoid too many letters, the superscripts in the tables depict significant differences between treatments within each type of feed.

## 3. Results

### 3.1. Rumen fermentation characteristics of the basal feeds

The results on TGP, CH_4_ production, DM and aNDFom degradability (dDM and dNDF, respectively) are reported in Table 1. The highest TGP was produced during fermentation of MS-CTR (152 vs. 122 mL/g DM, for MS-CTR and GS-CTR, respectively, *p* = 0.038) and the same tendency was observed for CH_4_ production (MS-CTR: 10.7 and GS-CTR: 4.36 mL CH_4_/g DM; *p* = 0.055). Both basal feeds had a dDM around 71–72%, although GS-CTR had higher dNDF than MS-CTR (51.4 vs. 67.5%, for MS and GS, respectively, *p* < 0.001).

**Table 1:** Feed degradability, gas production, CH_4_ production, NH_3_ concentration, and ruminal fermentation parameters.

Feed	Additive	Dose	dDM %	dNDF %	TGP mL/g DM	TGP mL/g dDM	CH_4_ mL/g DM	CH_4_ mL/g dDM	pH	NH_3_ mM	VFA mmol/L	Acetic %VFA	Propionic %VFA	Butyric %VFA	Valeric %VFA	Caproic %VFA	Iso-butyric %VFA	Iso-valeric %VFA
Maize Silage	MS	0	70.9^a^	51.4^a^	152^a^	215a^bc^	10.7^a^	15.1^a^	6.80	11.8^bc^	69.1	69.3	16.5^b^	10.2^abc^	1.10^bc^	0.436^a^	0.95^ab^	1.50^abc^
	CAT	1.5	70.1^a^	50.9^a^	155^a^	221^abc^	10.7^a^	15.2^a^	6.76	11.6^bcd^	64.9	69.5	16.5^b^	10.0^abc^	1.10^bc^	0.437^a^	0.956^ab^	1.48^bc^
		3	71.6^a^	51.8^a^	155^a^	217^abc^	11.2^a^	15.7^a^	6.73	11.9^bc^	65.3	69.6	16.4^b^	10.0^abc^	1.09^c^	0.422^a^	0.944^ab^	1.47^bc^
		6	67.4^a^	48.4^a^	155^a^	230^ab^	10.3^a^	15.4^a^	6.73	11.5^bcd^	64.7	70.5	16.1^b^	9.53^bc^	1.08^cd^	0.419^a^	0.918^b^	1.43^bc^
	QUE	1.5	67.2^a^	48.9^a^	153^a^	228^ab^	10.8^a^	16.1^a^	6.73	11.3^cd^	63.4	69.1	16.8^b^	10.4^abc^	1.08^cd^	0.382^ab^	0.898^b^	1.38^c^
		3	65.3^a^	41.2^a^	129^b^	198^bc^	5.25^b^	8.17^b^	6.77	10.5^de^	64.0	67.7	18.7^ab^	10.5^abc^	1.02^d^	0.239^bc^	0.757^c^	1.09^d^
		6	54.7^b^	14.0^b^	102^c^	187^c^	1.50^b^	2.88^c^	6.71	9.78^e^	61.4	68.4	20.1^a^	9.00^c^	0.909^e^	0.214^c^	0.584^d^	0.789^e^
	SALA	1.5	68.9^a^	48.7^a^	150^ab^	217^abc^	11.1^a^	16.1^a^	6.80	11.9^bc^	68.8	69.2	16.4^b^	10.4^abc^	1.10^bc^	0.434^a^	0.956^ab^	1.5^abc^
		3	70.0^a^	48.5^a^	154^a^	225^ab^	10.9^a^	15.9^a^	6.56	11.9^bc^	62.8	69.2	16.3^b^	10.5^abc^	1.13^abc^	0.452^a^	0.968^ab^	1.51^abc^
		6	67.6^a^	43.3^a^	150^ab^	215^abc^	10.5^a^	15.6^a^	6.80	12.1^bc^	68.0	69.8	15.8^b^	10.3^abc^	1.11^bc^	0.459^a^	0.959^ab^	1.50^abc^
	TAN	1.5	67.7^a^	52.0^a^	159^a^	235^a^	12.2^a^	18.0^a^	6.76	13.6^a^	68.0	68.6	16.6^b^	10.4^abc^	1.20^a^	0.469^a^	1.03^a^	1.68^a^
		3	67.5^a^	51.1^a^	154^a^	229^ab^	11.2^a^	16.6^a^	6.75	13.5^a^	66.3	67.9	17.1^ab^	10.8^ab^	1.19^a^	0.455^a^	0.997^ab^	1.59^abc^
		6	66.2^a^	48.3^a^	150^ab^	227^ab^	10.3^a^	16.0^a^	6.74	12.7^ab^	63.9	66.3	18.6^ab^	11.2^a^	1.16^ab^	0.422^a^	0.931^ab^	1.44^bc^
	SE		1.99	2.37	5.68	11.8	1.45	2.23	0.082	0.862	4.17	0.612	0.803	0.611	0.029	0.029	0.04	0.054
Grass Silage	GS	0	71.9^a^	67.5^a^	122^ab^	170^cd^	4.36^a^	6.10^b^	6.85	18.5^ab^	66.5	66.5^e^	19.8^ab^	8.87^cd^	1.65^bc^	0.308^cd^	1.12^b^	1.80^b^
	CAT	1.5	70.7^ab^	65.5^ab^	121^ab^	171^cd^	4.38^a^	6.23^ab^	6.88	17.7^cd^	67.9	67.3^cd^	19.3^cde^	8.72^cd^	1.59^de^	0.303^de^	1.07^c^	1.69^c^
		3	70.3^ab^	65.3^ab^	121^ab^	172^cd^	4.75^a^	6.79^ab^	6.86	17.8^cd^	65.7	67.6^c^	19.1^de^	8.63^d^	1.57^de^	0.297^def^	1.07^c^	1.68^cd^
		6	70.6^ab^	65.9^ab^	121^ab^	172^cd^	4.24^a^	6.00^b^	6.85	17.6^cd^	70.7	68.4^b^	18.9^ef^	8.22^e^	1.51^f^	0.282^ef^	1.03^d^	1.60^e^
	QUE	1.5	70.0^ab^	65.1^ab^	121^ab^	173^bcd^	4.53^a^	6.48^ab^	6.80	17.4^d^	66.3	67.1^d^	19.8^a^	8.56^d^	1.54^ef^	0.276^fg^	1.03^d^	1.62^de^
		3	65.3^cd^	59.6^c^	110^cd^	168^cd^	2.49^b^	3.81^c^	6.87	15.8^e^	66.5	68.3^b^	19.5^abcd^	8.08^e^	1.49^f^	0.259^g^	0.945^e^	1.47^f^
		6	58.1^e^	46.9^d^	108^d^	190^a^	1.87^b^	3.24^c^	6.86	15.0^f^	63.5	68.9^a^	16.5^g^	10.4^a^	1.63^cd^	0.347^ab^	0.903^f^	1.34^g^
	SALA	1.5	71.2^ab^	65.3^ab^	118^abc^	166^d^	4.47^a^	6.28^ab^	6.88	17.8^cd^	65.4	67.2^d^	19.3^cde^	8.81^cd^	1.59^cde^	0.304^de^	1.08^c^	1.71^c^
		3	69.0^abc^	62.6^bc^	121^ab^	175^abcd^	4.13^a^	6.20^ab^	6.85	17.7^cd^	65.9	67.4^cd^	19.1^de^	8.77^cd^	1.60^cd^	0.307^cd^	1.07^c^	1.68^c^
		6	68.7^abc^	60.2^c^	113^bcd^	165^d^	4.36^a^	6.38^ab^	6.87	17.7^cd^	64.1	67.6^c^	18.7^f^	8.99^c^	1.61^cd^	0.313^cd^	1.08^c^	1.70^c^
	TAN	1.5	69.4^abc^	66.4^ab^	125^a^	180^abcd^	5.40^a^	7.77^ab^	6.87	18.8^a^	66.0	66.0^f^	19.6^abc^	9.38^b^	1.69^ab^	0.329^bc^	1.16^a^	1.85^a^
		3	67.4^bcd^	63.1^bc^	123^a^	183^ab^c	5.26^a^	7.69^ab^	6.84	18.6^ab^	65.4	66.0^f^	19.3^cde^	9.59^b^	1.72^a^	0.357^a^	1.15^ab^	1.83^ab^
		6	64.0^d^	60.3^c^	121^ab^	189^ab^	5.09^a^	7.96^a^	6.9	18.1^bc^	63.5	66.1^ef^	19.4^bcd^	9.57^b^	1.69^ab^	0.339^ab^	1.13^ab^	1.78^b^
	SE		0.765	0.681	4.38	6.86	0.542	0.794	0.041	0.22	1.8	0.255	0.442	0.183	0.015	0.010	0.005	0.01
	*p*-value																	
	Feed		0.529	<0.001	0.038	0.065	0.055	0.062	0.222	0.020	0.980	0.011	0.094	0.156	<0.001	0.019	0.050	0.022
	Additive		<0.001	<0.001	<0.001	0.004	<0.001	<0.001	0.891	<0.001	0.098	<0.001	0.112	0.008	<0.001	<0.001	<0.001	<0.001
	Feed × Additive		0.700	0.011	0.005	0.039	0.001	0.002	0.866	<0.001	0.062	0.015	0.009	0.817	<0.001	<0.001	0.001	<0.001
MS	Additive		0.001	<0.001	<0.001	0.004	<0.001	<0.001	0.870	<0.001	0.165	0.004	0.001	0.009	<0.001	<0.001	<0.001	<0.001
MS	Level(Additive)		0.010	<0.001	<0.001	0.026	<0.001	<0.001	0.297	0.021	0.571	0.233	0.029	0.024	<0.001	0.034	<0.001	<0.001
GS	Additive		<0.001	<0.001	<0.001	<0.001	<0.001	<0.001	0.878	<0.001	0.045	<0.001	<0.001	<0.001	<0.001	<0.001	<0.001	<0.001
GS	Level(Additive)		<0.001	<0.001	0.002	0.003	<0.001	<0.001	0.734	<0.001	0.129	<0.001	<0.001	<0.001	<0.001	<0.001	<0.001	<0.001

dDM, dry matter degradability; dNDF, neutral detergent fiber degradability; TGP, total gas production; VFA, volatile fatty acids; MS, maize silage; CAT, catechin; QUE, quercetin; SALA, salicylic acid; TAN, tannic acid; SE, standard error; GS, grass silage.

^a,b,c^ Different letters on the same column within the feed correspond to different least squares means after Tukey’s adjustment (*p* < 0.05).

As shown in Table 1, although the total VFA concentration (mmol/L) in the fluid post-fermentation was of similar magnitude with both basal feeds, significant differences were observed in relation to the composition of produced VFAs, where the % of total VFA was higher with MS-CTR for acetic (69.3 vs. 66.5% for MS and GS, *p* = 0.011), and caproic (0.44 vs. 0.31% for MS and GS, *p* = 0.019) acids as compared to GS-CTR, and the reverse was true for iso-butyric (0.95 vs. 1.12% for MS and GS, *p* = 0.050), iso-valeric (1.50 vs. 1.80% for MS and GS, *p* = 0.022) and valeric (1.10 vs. 1.65% for MS and GS, *p* < 0.001) acids. No significant differences were observed for propionic (16.5 vs. 19.8% for MS and GS) and butyric acids (10.2 vs. 8.87% for MS and GS). The fermentation of MS-CTR resulted in lower NH_3_ concentrations in the fermented fluid by the end of fermentation as compared to GS-CTR (11.8 vs. 18.5 mM for MS and GS, *p* = 0.020).

### 3.2. Impact of PSMs using maize silage as substrate

#### 3.2.1. Feed degradability, TGP, and CH_4_ production using maize silage as substrate

**Figure 1:**
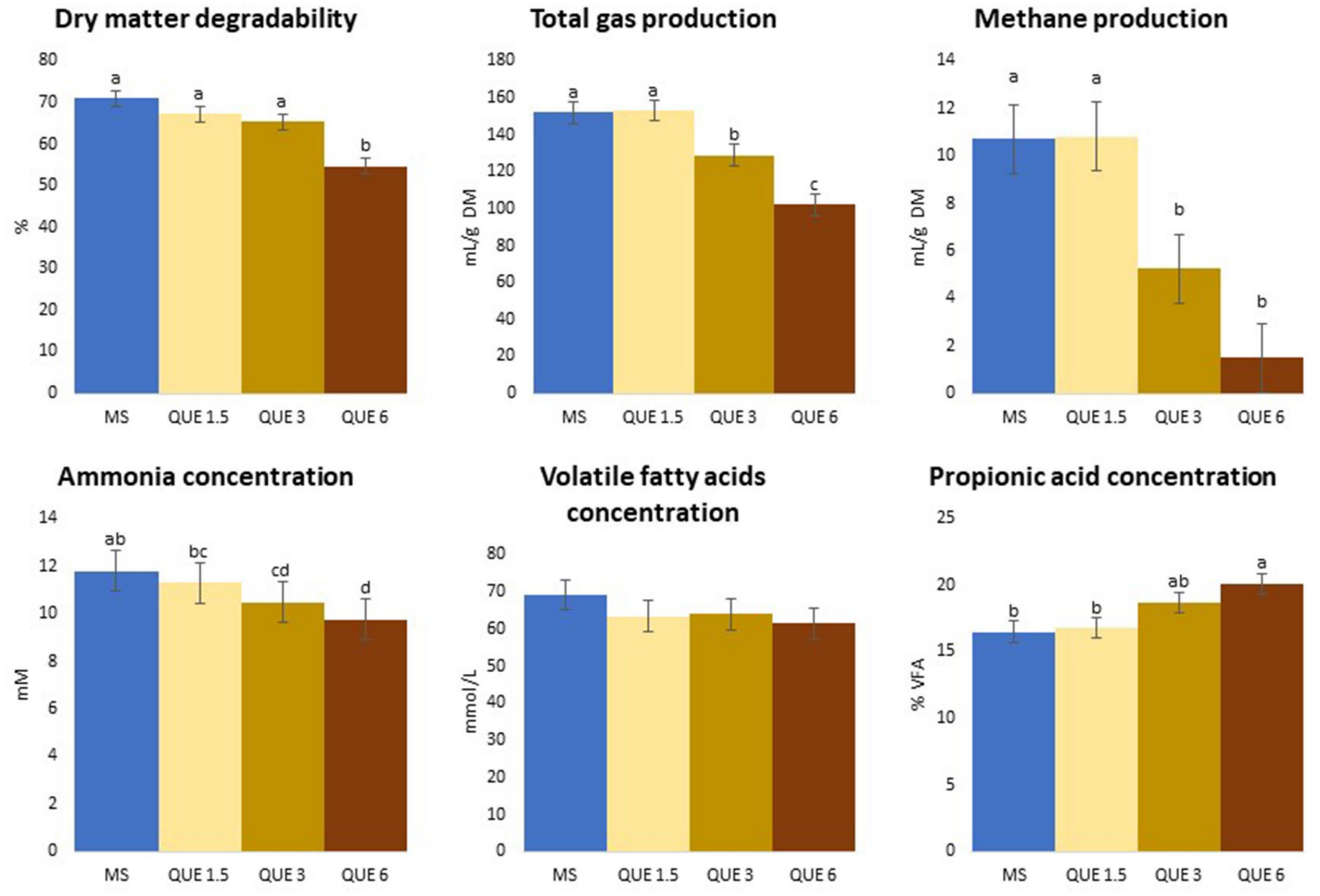
Effect of the dose of inclusion (1.5, 3, and 6% of feed dry matter) of quercetin (QUE) on degradability, total gas and methane productions, ammonia, total volatile fatty acids (VFA) and propionic acids concentrations with maize silage (MS) as basal feed.

When MS was used as feed substrate, increasing dose of QUE caused a linear reduction of both dDM (*p* = 0.022) and dNDF (*p* = 0.002), however, the differences relative to MS-CTR became significant only at the highest inclusion rate, where the overall reduction in dDM was 23% compared to MS-CTR. With increasing dose of QUE there was also a linear reduction of TGP (mL/g DM; *p* = 0.005) and CH_4_ production (mL/g DM; *p* = 0.004; and mL/g degraded DM; *p* = 0.005), Tables 1, 2 and Figure 1. QUE significantly reduced CH_4_ production (mL/g DM) at the inclusion doses of 3 and 6%, with a reduction of 51 and 86%, respectively, compared to MS-CTR. No other additive caused significant changes of the above-mentioned parameters.

**Table 2:** *p*-value of the linear and quadratic effects of the dose of the plant secondary metabolites for the variables studied.

Feed	Additive	dDM %	dNDF %	TGP mL/g DM	TGP mL/g dDM	CH_4_ mL/g DM	CH_4_ mL/g dDM	pH	NH_3_ mM	VFA mmol/L	Acetic %VFA	Propionic %VFA	Butyric %VFA	Valeric %VFA	Caproic %VFA	Iso-butyric %VFA	Iso-valeric %VFA
Maize Silage	Linear effect																
	CAT	0.061	0.041	0.293	0.027	0.547	0.722	0.032	0.159	0.361	0.011	0.014	0.012	0.324	0.095	0.022	0.036
	QUE	0.022	0.002	0.005	0.069	0.004	0.005	0.226	0.007	0.096	0.382	0.039	0.031	0.001	0.005	0.001	0.001
	SALA	0.064	<0.001	0.060	0.966	0.716	0.782	0.916	0.236	0.676	0.016	0.003	0.484	0.513	0.017	0.776	0.984
	TAN	0.007	0.119	0.501	0.534	0.389	0.906	0.206	0.169	0.026	0.017	0.033	0.105	0.087	0.566	0.178	0.093
	Quadratic effect																
	CAT	0.154	0.118	0.365	0.306	0.240	0.637	0.116	0.563	0.462	0.323	0.455	0.295	0.598	0.770	0.150	0.613
	QUE	0.573	0.126	0.373	0.690	0.798	0.599	0.974	0.374	0.394	0.415	0.997	0.055	0.242	0.153	0.776	0.735
	SALA	0.467	0.214	0.103	0.043	0.539	0.365	0.255	0.859	0.272	0.048	0.244	0.259	0.290	0.904	0.324	0.984
	TAN	0.682	0.354	0.406	0.235	0.223	0.167	0.481	0.008	0.951	0.857	0.385	0.957	0.020	0.361	0.034	0.023
Grass Silage	Linear effect																
	CAT	0.406	0.235	0.854	0.766	0.538	0.767	0.743	0.008	0.229	<0.001	0.002	0.002	0.003	0.008	<0.001	<0.001
	QUE	<0.001	<0.001	0.002	0.005	<0.001	<0.001	0.729	<0.001	0.112	<0.001	<0.001	<0.001	0.963	0.008	<0.001	<0.001
	SALA	0.013	0.002	0.099	0.462	0.724	0.584	0.964	0.013	0.059	<0.001	0.002	0.137	0.052	0.030	0.005	0.007
	TAN	0.001	0.004	0.288	0.019	0.426	0.123	0.094	0.071	0.028	0.123	0.055	0.005	0.007	0.002	0.793	0.147
	Quadratic effect																
	CAT	0.380	0.099	0.657	0.818	0.041	0.038	0.530	0.039	0.403	0.009	0.027	0.316	0.405	0.581	0.026	0.017
	QUE	0.172	<0.001	0.079	0.018	0.086	0.087	0.905	0.008	0.332	0.034	<0.001	<0.001	0.003	0.002	0.001	0.005
	SALA	0.243	0.156	0.547	0.312	0.436	0.947	0.997	0.016	0.772	0.007	0.197	0.074	0.025	0.064	0.006	0.007
	TAN	0.467	0.775	0.202	0.251	0.220	0.220	0.185	0.110	0.547	0.051	0.137	0.010	0.004	0.001	0.019	0.023

dDM, dry matter degradability; dNDF, neutral detergent fiber degradability; TGP, total gas production; VFA, volatile fatty acids; CAT, catechin; QUE, quercetin; SALA, salicylic acid; TAN, tannic acid.

#### 3.2.2. Rumen fermentation parameters using maize silage as substrate

As shown in Tables 1, 2 and Figure 1, the pH, total concentration of VFA and percentages of acetic and butyric acids in the fermented liquid post-fermentation were unaffected by addition of any of the additives at any dose.

However, the concentration of propionic acid in the fermented liquid, which is produced in a hydrogen consuming pathway, was linearly increased by QUE (*p* = 0.039) consistent with the depression of CH_4_ production, and at the 6% dose, propionic acid concentration became significantly higher compared to MS-CTR (20.1 vs. 16.5%). Proportions of the minor VFA’s, iso-butyric, iso-valeric, valeric, and caproic acid, were linearly reduced by QUE and, for all of these, the values observed at 3 and 6% of QUE inclusion were significantly lower than MS-CTR.

Consistent with the decrease in the two iso-acids, derived in the rumen from branched chained amino acid degradation, the NH_3_ concentration was also linearly reduced by QUE (*p* = 0.007) and at 3 and 6% the reduction became significantly different from MS-CTR (10.5 and 9.78 vs. 11.8 mM, respectively), while there was a quadratic effect of increased TAN inclusion (*p* = 0.008) due to increased NH_3_ concentration at 1.5 and 3% TAN only.

### 3.3. Impact of PSMs using grass silage as substrate

#### 3.3.1. Feed degradability, TGP, and CH_4_ production using grass silage as substrate

When GS was used as feed substrate, dDM was linearly reduced by QUE and TAN (*p* < 0.001 and *p* = 0.001, respectively) and at doses of 3 and 6% the decrease in dDM became significantly different from GS-CTR (65.3 and 58.1, respectively, for QUE, and 67.4 and 64.0, respectively, for TAN vs. 71.9% for GS-CTR). Furthermore, QUE decreased dNDF at the two highest but not the lowest dose relative to GS-CTR (quadratic effect; *p* < 0.001), while a linear depression of dNDF was induced by increasing doses of SALA and TAN (*p* = 0.002 and *p* = 0.004, respectively), hence for all 3 PSMs the depressions at the 3 and 6% doses reduced dNDF to significantly lower levels than for GS-CTR (59.6 and 46.9, respectively, for QUE; 62.6 and 60.2, respectively, for SALA; 63.1 and 60.3 for TAN, vs. 67.5% for GS-CTR).

**Figure 2:**
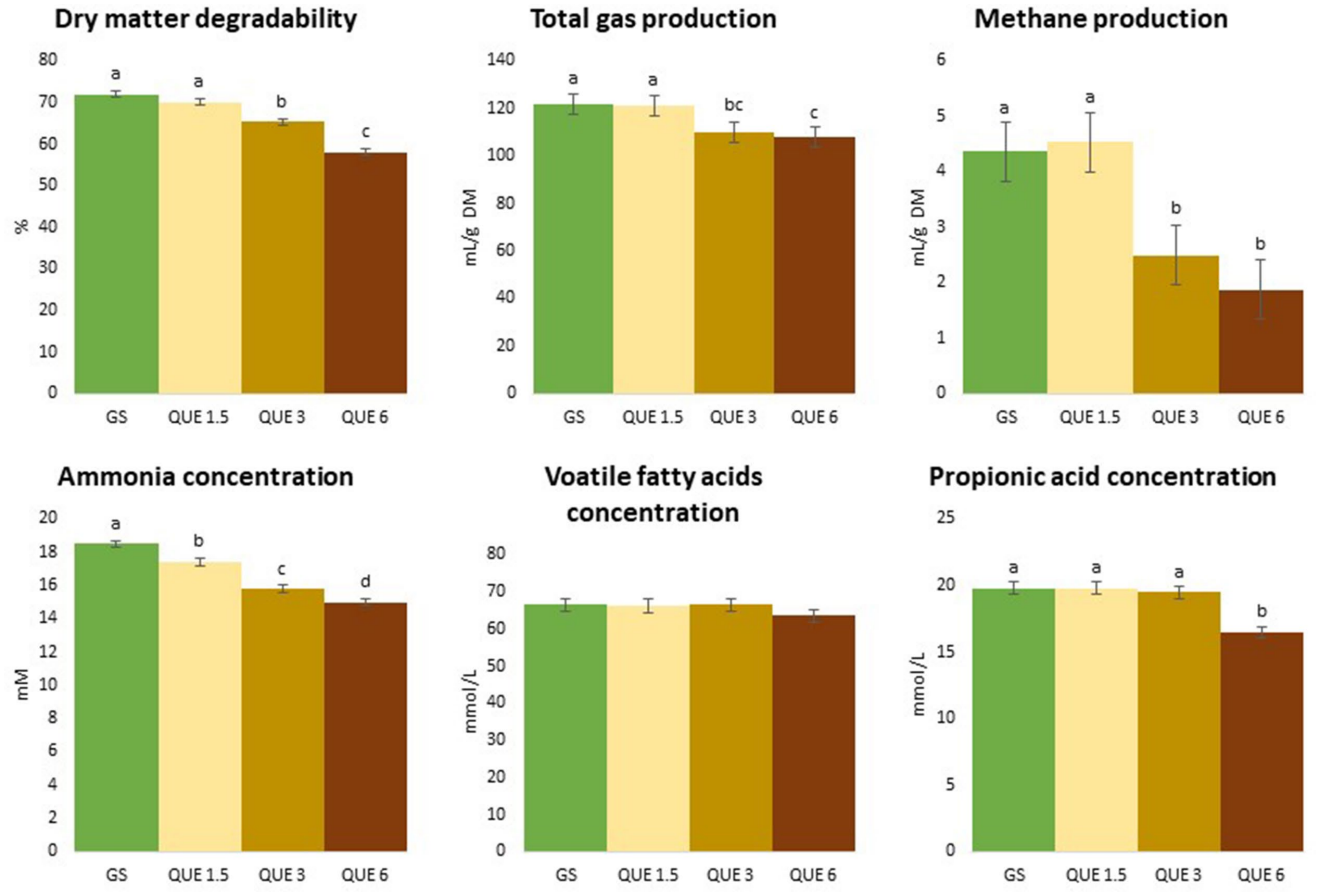
Effect of the dose of inclusion (1.5, 3, and 6% of feed dry matter) of quercetin (QUE) on degradability, total gas and methane productions, ammonia, total volatile fatty acids (VFA) and propionic acids concentrations with grass silage (GS) as basal feed.

The TGP and CH_4_ production (mL/g DM for both) were linearly reduced by QUE (*p* = 0.002 and *p* < 0.001), and for CH_4_ production the reductions at the 3 and 6% doses were 43 and 58%, respectively, compared to GS-CTR, Tables 1, 2 and Figure 2. Due to the simultaneous reduction of dDM, TGP when expressed relative to degraded DM (mL/g dDM) became significantly increased compared to GS-CTR with addition of QUE and TAN only at the highest dose (6%). Similarly, TAN at the highest dose reduced dDM more than CH_4_ production relative to GS-CTR and CH_4_ production per degraded DM was consequently increased (7.96 vs. 6.10 mL/g dDM for GS-CTR). However, QUE reduced CH_4_ production substantially more than dDM and still linearly reduced (*p* < 0.001) CH_4_ production per g degraded DM up to 46.9% at the highest (6%) dose. None of the other additives reduced TGP or CH_4_ production.

#### 3.3.2. Rumen fermentation parameters using grass silage as substrate

Similarly, to when MS was used as basal feed, the pH and total VFA concentration in the fermented liquid post-fermentation were unaffected by the additives at any dose, as shown in Tables 1, 2, although a linear reduction of total VFA was observed with increasing dose of TAN (*p* = 0.028).

The proportion of acetic acid in total VFA was affected in a quadratic fashion with increasing dose of CAT (*p* = 0.009), QUE (*p* = 0.034), and SALA (*p* = 0.007) with effects levelling off at higher doses and differences became significant relative to GS-CTR already at the 1.5% dose. Reversely, percentages of propionic and iso-butyric acids were reduced with increasing dose of CAT (*p* = 0.027 and *p* = 0.026, respectively, for quadratic effects), QUE (*p* < 0.001 and *p* = 0.001; respectively for quadratic effects), and SALA (*p* = 0.002 for linear effect and *p* = 0.006 for quadratic effect, respectively), and the reductions for all three additives became significantly different relative to GS-CTR already at the lowest dose of 1.5%.

The additives had differential and inconsistent patterns of effects on proportions of butyric, iso-valeric, valeric and caproic acids in total VFA.

Butyric acid proportion in total VFA was linearly reduced by CAT (*p* = 0.002) and became significantly lower than GS-CTR at the highest (6%) dose; QUE reduced the proportion of butyric acid relative to GS-CTR at the 3% dose but the proportion was increased at the highest dose (8.08, 10.4 vs. 8.87% for GS-CTR, respectively; *p* < 0.001 for quadratic effect); while butyric acid proportions increased with increasing dose of TAN (*p* = 0.010 for quadratic effect).

The iso-valeric proportion was reduced in a quadratic fashion by CAT (*p* = 0.017), QUE (*p* = 0.005), and SALA (*p* = 0.007), but increased by TAN relative to GS-CTR. The valeric acid proportions were increased by CAT and TAN (*p* = 0.003 for linear and *p* = 0.004 for quadratic effects, respectively), but reduced by QUE (*p* = 0.003 for quadratic effect).

CAT linearly reduced (*p* = 0.008) the proportion of caproic acid in total VFA (significantly different from GS-CTR at the 6% dose), whereas the proportion was increased by TAN (*p* = 0.001 for quadratic effect), and decreased by QUE at the 1.5 and 3% doses only but increased at the 6% dose relative to GS-CTR (0.276, 0.259, and 0.347, respectively, vs. 0.308 for GS; *p* = 0.002 for quadratic effect).

The NH_3_ concentration (mM) was reduced in a quadratic fashion with increasing dose of CAT (*p* = 0.039), QUE (*p* = 0.008), and SALA (*p* = 0.016). Already at 1.5% of inclusion, CAT, QUE, and SALA had significantly lowered NH_3_ concentrations in the fermented liquid post-fermentation compared to GS-CTR, and the greatest reduction of 19% was observed with QUE at a dose of 6%, Tables 1, 2.

## 4. Discussion

With this *in vitro* experiment that simulates ruminal fermentation, the first of two aims were to investigate the individual effects of four PSMs, CAT, QUE, SALA, and TAN, using three inclusion doses (1.5, 3 and 6% w/w DM) and two basal feeds (MS and GS) when co-incubated in buffered rumen fluid. In a previous experiment (21), we observed potential anti-methanogenic effects of these additives, therefore we investigated whether the effects of these compounds were dose and substrate dependent. Since several reviews (31–33) conclude that PSMs can have different effects depending on the composition of the basal feed and the dose, we chose to use two different feeds, MS and GS, as substrates and three doses.

### 4.1. Rumen fermentation characteristics of the basal feeds

In GS, NDF dominates the carbohydrate fraction and starch content is very low, whereas MS has a higher starch concentration (24). In this study, MS had a similar dDM and a lower dNDF but, due to the higher starch content, resulted in a higher TGP (mL/g DM) compared to GS. This difference in TGP between MS and GS is in agreement with the results of García-Rodriguez et al. (34), who found that after 96 h of incubation *in vitro*, MS and GS had a mean difference of 24% in TGP.

Regarding CH_4_ production, in the present study we registered a higher CH_4_ production with MS compared to GS, which is also in line with the higher concentration of acetic acid. Although, Staerfl et al. (35) found a higher CH_4_ yield in fattening bulls when fed MS compared to GS, most *in vivo* studies comparing the two forages generally have shown the opposite results, where GS diets generate more CH_4_ than MS diets (24, 36, 37). According to Hart et al. (38), a higher ratio of MS to GS in the diet resulted in a lower CH_4_ yield from dairy cows due to the higher amount of starch, which favors hydrogen consuming fermentation pathways leading to formation of propionic acid, thereby diverting hydrogen away from CH_4_ production (39). Feeding diets high in NDF on the other hand can favor net-hydrogen producing fermentation pathways leading to more acetic acid formation. This difference between the results obtained *in vitro* and those obtained *in vivo* could probably be explained by adaptation of the rumen microbial population over time to diets *in vivo*, resulting in changes in microbe specific fermentation pathways, which will not happen over the short time duration of *in vitro* experiments. The *in vitro* experiments generally lasted 48 h, which must be insufficient time for the microbial population of the rumen fluid donor cows to adapt to the new “diet.”

Generally higher concentration of NH_3_ was produced when GS was used as substrate, which is in accordance with the fact that GS has higher CP content compared to MS (179 vs. 77.7 g/kg).

### 4.2. Impact of PSMs on feed degradability, TGP, CH_4_ production, and rumen fermentation parameters

Among the additives tested, QUE induced specific and dramatic reductions in CH_4_ production, and this could not simply be ascribed to an overall suppression of fermentation, since the reduction in CH_4_ production was more extensive than the observed reduction in feed degradability and TGP. Yet, the reduction in dDM and dNDF did not reduce overall VFA concentration.

The reductions in CH_4_ production induced were consistent on both feeds and there was a linear reduction with increased dose of QUE. In the present study, the ability of QUE to reduce CH_4_ production was observed in combination with both types of substrates, and at the highest dose (6%) of QUE the CH_4_ production was reduced to similar levels of 2.9 (MS) to 3.2 (GS) mL/g dDM. However, the quantitative reduction was substantially higher with MS than GS as substrate (80.6 vs. 57.1%) due to the higher intrinsic CH_4_ production during fermentation of MS (15.1 mL/g dDM) compared to GS (6.1 mL/g dDM). Results from previous *in vitro* studies on anti-methanogenic effect of QUE have been consistent. Both *in vitro* studies, Oskoueian et al. (19) and Sinz et al. (17), found that QUE inhibited CH_4_ production. In the study of Oskoueian et al. (19), QUE induced the anti-methanogenic effect at the dose 4.5–5% of the substrate DM. Addition of QUE at 4.5% of DM to a mixture of guinea grass and concentrate (60:40) induced a reduction of 27.9% in CH_4_ (mL/g DM) combined with an increase in TGP compared to the control. Sinz et al. (17) performed *in vitro* dose–response study with the concentration of QUE at 0.05, 0.5 and 5% of the substrate DM. At all doses QUE was an effective anti-methanogenic additive (17), 5% of QUE caused a 22.8% reduction in CH_4_ production (mL) *in vitro*. In our study the anti-methanogenic effect of QUE linearly diminished between the dose of 3 to 1.5% of the substrate DM for MS as basal feed and between 3 to 6% for GS as basal feed. In the study of Sinz et al. (17) the anti-methanogenic effect of QUE was significant even at 0.5% of the substrate DM, which is not consistent to our study. The different outcomes observed can be due to the characteristics of the substrates used and their interaction with QUE. This aspect has been observed also with other additives, both *in vitro* and *in vivo*. For example an *in vitro* study of Castro-Montoya et al. (40) tested seven feed additives of different types and with different mode of action on concentrate, GS, MS and a mixture of the three of them, finding different responses of each additive on CH_4_ mitigation depending on the substrates. The new commercially available additive 3-nitrooxypropanol (3-NOP) is another example of an additive, where the anti-methanogenic potential is influenced by the basal diet. The study of van Gastelen et al. (41) tested the CH_4_ mitigation potential of 3-NOP on dairy cows by adding it to a maize silage-based diet, a grass silage-based diet, or mix of them. Similarly, to what we observed with QUE, 3-NOP decreased CH_4_ yield from cows fed either of the diets, however the reduction was smaller for grass silage-based diet compared with both the mixed and maize silage-based diet. One possible explanation for the greater efficacy of QUE with MS rather than GS as a substrate could be linked to its action on protozoa. In the rumen, certain methanogens have a symbiotic relationship with the protozoa (42), and Lengowski et al. (43) observed a greater abundance of protozoa in dairy cows that were fed a diet based on MS compared to a GS-based diet. In an *in vitro* study, Kim et al. (44) found that a reduction in the population of protozoa was associated with reduced CH_4_ production when different flavonoid-rich plant extracts were added to feed substrate. Thus, being a flavonoid, QUE may have reduced CH_4_ more efficiently with MS as the substrate due to suppression of an otherwise more extensive growth and/or metabolism of protozoa and hence methanogen populations, when MS rather than GS was provided as a substrate for fermentation. The anti-microbial effect of flavonoids could be due to inhibition of bacterial cytoplasmic membrane function, their cell wall synthesis, or through inhibition of their nucleic acid synthesis (19, 45). Thus, reduced protozoa, protozoa-associated methanogens and free methanogens are likely explanations for the reduction on CH_4_ production induced by QUE. Other tested PSMs in the present study did not have specific suppressive actions on CH_4_ production, but they did interfere with ruminal feed fermentation in other ways.

The observed decrease in dDM and/or dNDF of either of the two feed substrates with addition of increasing doses of TAN, can be ascribed to the generally recognized ability of tannins to bind to proteins and other feed components, thereby decreasing microbial access and ruminal degradability (46). However Getachew et al. (47, 48), in contrast to our results, found no impact on *in vitro* degradability when TAN was added at concentrations of up to 10% of substrate DM. To the best of our knowledge, no one has ever previously studied how SALA influences rumen fermentation. For SALA and TAN, but not QUE, depressions in dDM were associated with linear decreases in total VFA concentrations, although concentrations never differed significantly from those obtained, when the basal feeds were fermented without any additive. Changes in dDM were generally associated with changes in dNDF, but neither of the two were necessarily related to changes in TGP, CH_4_ production or total VFA concentration. All the additives induced changes in the relative proportions of the individual VFAs. In this context it is relevant to keep in mind that in the absence of changes in overall production, a change in the proportion of a single VFA will inevitably result in changes to the percentages of the other VFAs.

The type of basal feed did not influence the total concentration of VFA, but major changes occurred in proportions of the individual VFA. There were consistent effects across PSMs of increasing doses on proportions of acetic and propionic acids in the fermented inoculum post-fermentation, but quantitative changes were modest. Among the PSMs, QUE was the only one that induced a linear increase in propionic acid proportions although only when added to MS. During rumen fermentation, pathways resulting in propionic acid formation act as hydrogen sinks and hence competes with CH_4_ formation for hydrogen. Pathways leading to formation of acetic and butyric acids on the other hand lead to net formation of hydrogen, which methanogens can subsequently use to convert CO_2_ to CH_4_ (49). Thus, the observed increase of propionic acid is consistent with an anti-methanogenic effect of QUE, since excess hydrogen following inhibition of CH_4_ formation would give rise to channeling of excess hydrogen into alternative pathways.

The same effect of QUE on propionic acid was not observed with GS as feed substrate, and unexpectedly QUE increased the proportion of acetic acid with GS as a substrate. We have no immediate explanation for these contradictory results depending on the nature of the basal feed. Acetic acid originates mainly from fiber degradation, and it is odd to observe an increase in acetic acid proportion coinciding with a reduction in NDF degradability. However, others have also reported that inhibition of methanogenesis in some cases results in an increased proportion of propionic acid at the expense of acetic acids (26, 49).

The second aim of this experiment was to evaluate whether the studied PSMs had the ability to influence NH_3_ formation in the rumen as an indicator of changes in N-metabolism, which is an important concern in terms of optimizing N-utilization and minimizing N-excretion from the animal. Increased doses of QUE when co-incubated with both feeds, and of CAT and SALA when co-incubated with GS only, reduced concentrations of NH_3_ in the fermented liquid. Such a reduction of NH_3_ concentration could reflect either a reduced feed protein degradation or an increased microbial protein synthesis. Since QUE, CAT, and SALA all had a suppressive effect on feed degradability, it is unlikely that microbial protein synthesis was enhanced by these PSMs. Therefore, the most plausible explanation for the observed reductions of NH_3_ concentrations is a suppression of protein degradation. The simultaneous changes in iso-butyric, valeric, and iso-valeric acids concentrations seem to support this, since these VFAs are byproducts from branched-chained amino acid deamination. Our study showed that flavonoids, such as QUE and CAT, and phenolic acids, such as SALA, are able to induce effects on *N* metabolism similar to those of tannins. This can protect feed protein from ruminal degradation, reduce NH_3_ absorption and potentially decrease *N* losses via urinary excretion.

## 5. Conclusion

Among the tested compounds, only the flavonoid QUE could inhibit CH_4_ emission from rumen feed fermentation, but the dose-dependent magnitude of change was more pronounced when MS was used as the feed substrate compared to GS. Both of the tested flavonoids, CAT and particularly QUE as well as the phenolic acid SALA, but not the hydrolysable tannin TAN, had more or less extensive suppressive effects on rumen metabolic pathways leading to NH_3_ formation, and when manifested depending on the basal feed, this could apparently be ascribed to suppression of bacterial amino acid catabolism. These findings encourage to further *in vivo* studies to verify whether QUE at a dietary inclusion rate of 3% in DM can reduce CH_4_ emission also in dairy cows, which would open for its possible use as a natural and safe feed additive to suppress both ruminal CH_4_ formation and protein degradation without affecting overall bacterial VFA production.

## Data availability statement

The raw data supporting the conclusions of this article will be made available by the authors, without undue reservation.

## Ethics statement

The animal study was approved by Danish Ministry of Environment and Food. The study was conducted in accordance with the local legislation and institutional requirements.

## Author contributions

MB: Conceptualization, Data curation, Formal analysis, Investigation, Methodology, Validation, Visualization, Writing – original draft. MN: Conceptualization, Data curation, Formal analysis, Funding acquisition, Investigation, Methodology, Project administration, Resources, Supervision, Validation, Writing – review & editing. NN: Conceptualization, Data curation, Formal analysis, Funding acquisition, Investigation, Methodology, Project administration, Resources, Supervision, Validation, Visualization, Writing – original draft, Writing – review & editing.
